# 

**Published:** 1896-07

**Authors:** 


**ESTABLISHED 1855.** **SUBSCRIPTION, $2.00.**

### ATLANTA MEDICAL AND SURGICAL JOURNAL.

VOLUME XIII. NEW SERIES. ATLANTA, GA., JULY, 1896. No. 5.

